# Aggressive low-density lipoprotein (LDL) lowering for primary prevention: still an elusive goal

**DOI:** 10.1186/s12944-024-02280-0

**Published:** 2024-09-06

**Authors:** Matin Sepehrinia, Reza Homayounfar, Mojtaba Farjam

**Affiliations:** 1 Noncommunicable Diseases Research Center, Fasa University of Medical Sciences, Fasa, Iran; 2 Faculty of Nutrition Sciences and Food Technology, National Nutrition and Food Technology Research Institute (WHO Collaborating Center), Shahid Beheshti University of Medical Sciences, Tehran, Iran

**Keywords:** Lipid, Cholesterol, Prevention, Dyslipidemia, Statin

## Abstract

Cardiovascular disease (CVD) is the leading cause of mortality globally. Low-density lipoprotein (LDL) plays an important role in CVD pathophysiology. Research has shown the safety and efficacy of keeping LDL at very low levels for CVD prevention. Therefore, experts recommend intense LDL-lowering approaches starting at young ages, promoting the mantras “the lower, the better” and “the earlier, the better.” This commentary discusses the challenges regarding applying aggressive LDL-lowering approaches in the general population, including pharmacological efficacy and side effects, the cost-effectiveness of interventions, and patient adherence to treatment regimens.

Cardiovascular diseases (CVDs) are considered the most common cause of mortality worldwide, accounting for approximately 19.8 million fatalities in 2022 [1]. High levels of low-density lipoprotein (LDL) play a substantial role in the formation of atherosclerosis. Therefore, LDL has been a target for preventing CVD. The latest prevailing guidelines recommend identifying high-risk individuals using risk assessment calculators and lowering their LDL levels through lipid-lowering drugs and lifestyle modifications [2]. Although this approach has been successful in reducing CVD mortality, the LDL levels in the general population are significantly higher than desirable levels [3]. Despite the remarkable efforts to lower LDL levels, approximately 70 million adults in the United States have an LDL level of more than 130 mg/dl [4]. Therefore, scientists are still looking for effective approaches to lower LDL levels for primary prevention.

Emerging evidence on the safety and beneficial effects of very low LDL levels has led to a shift in the paradigm of preventive cardiology. Lowering LDL showed promising effects on CVD, even in individuals with low LDL levels [5]. Recently, Michael E. Makover et al. [6] suggested early and aggressive reduction of LDL levels to prevent CVD in the general population. The mentioned study [6] proposed an LDL level below 55–70 mg/dl as the target for primary prevention based on the CVD risk profile, with a stricter LDL control for those with a higher CVD risk from a young age, ideally before the onset of atherosclerosis. While this strategy has the potential to revolutionize preventive cardiology, it has its challenges. In this article, we briefly point out the obstacles associated with early and aggressive LDL-lowering strategies.

Current recommendations from prevailing guidelines have highlighted the promising benefits of lifestyle modification in dyslipidemia management, such as healthy dietary habits and regular physical activity [2]. Lifestyle modification and consuming functional foods and nutraceuticals could effectively lower the LDL level and reduce the need for pharmacological interventions [7]. However, relying exclusively on lifestyle modification might not be enough to achieve the intended extremely low goal level of LDL among individuals with high LDL levels. Therefore, ensuring extremely low LDL levels in the general population necessitates a significant number of healthy individuals to use lipid-lowering medications over an extended period. As a result, it is essential to establish safe, efficient, and cost-effective preventive strategies. Despite the development of new generations of lipid-lowering medications, statins continue to be the first-line medication for primary and secondary prevention. High-intensity statins can only reduce LDL levels to 50% of the baseline, which might not be adequate for meeting the desired targets in the general population [8]. Consequently, additional medications like ezetimibe, bempedoic acid, or PCSK9 inhibitors may be required, alongside statins, to reach optimal targets. However, a meta-analysis has indicated that adding ezetimibe or PCSK9 inhibitors to statins did not improve cardiovascular outcomes in low-risk individuals [9]. Based on the International Lipid Expert Panel (ILEP) recommendation, bempedoic acid may be considered for primary prevention in high-risk patients who could not reach the desirable LDL level despite using the maximally tolerated dose of statins and ezetimibe (level B recommendation) [10]. However, evidence on using bempedoic acid for primary prevention in low-risk individuals is still scarce.

However, like all medications, lipid-lowering drugs have some adverse effects. While current evidence supports the idea that the benefits of lipid-lowering drugs outweigh the risks, prolonged use in a large portion of the population could magnify even rare adverse effects into significant public health concerns. Statin-associated muscle symptoms (SAMS) are the most prevalent side effect, affecting up to 29% of individuals taking statins [8]. Moreover, emerging research suggests that statins may increase the risk of new-onset diabetes and insulin resistance, which are fundamental contributors to cardiometabolic diseases [11]. Adverse effects of statins, particularly SAMS, are among the main reasons for statin non-adherence [12]; however, the prevalence of statin intolerance is usually overestimated. A recent meta-analysis showed that the prevalence of statin intolerance was 9.1% [13]. Also, PCSK9 inhibitors have certain limitations, including the need for subcutaneous injections, potential allergic reactions and myalgia at the injection site, and a high cost that may not be cost-effective for primary prevention at present [14, 15]. Inclisiran, a novel small interfering RNA inhibitor of PCSK9, increased the adherence by increasing the injection intervals to twice a year, but a recent study in the UK showed that Inclisiran is not cost-effective for primary prevention at the current cost [16]. Although bempedoic acid has demonstrated favorable outcomes in reducing major cardiac adverse events, HbA1c, and inflammatory markers, its administration is associated with an elevated risk of gout [17]. It is noteworthy that increased uric acid levels and gout symptoms are reversible upon discontinuation of the medication. The International Lipid Expert Panel holds the consensus that such adverse effects bear minimal clinical significance [10].

Michael E. Makover and colleagues have proposed initiating lipid-lowering treatment at a young age, ideally before the development of atherosclerosis, without relying on risk assessment tools [6]. Firstly, determining the appropriate age for commencing lipid-lowering medication is crucial. Secondly, another obstacle to the implication of the “the earlier, the better” strategy is that treatment adherence tends to be lower among younger individuals, particularly for primary prevention [18]. Thirdly, risk assessment currently serves as the foundation of primary prevention in existing guidelines. It facilitates shared decision-making and enhances lipid-lowering and anti-hypertensive treatments by evaluating overall CVD risk based on multiple risk factors, not solely lipid profiles [19].

Taken together, achieving extremely low LDL levels in the general population is still an elusive goal. Aggressive LDL-lowering treatment strategies should be defined concisely and their cost-effectiveness should be evaluated. Insurance companies must be willing to cover these strategies, and the public should be educated about the benefits of maintaining very low LDL levels to improve treatment adherence.

## Data Availability

No datasets were generated or analysed during the current study.

## Abbreviations

CVD: Cardiovascular disease
LDL: Low-density lipoprotein
ILEP: International Lipid Expert Panel
SAMS: Statin-associated muscle symptoms

## References

[1] Mensah GA, Fuster V, Murray CJ, Roth GA. Global burden of cardiovascular diseases and risks, 1990–2022. J Am Coll Cardiol. 2023;82(25):2350–473. 38092509 10.1016/j.jacc.2023.11.007 PMC7615984

[2] Arnett DK, Blumenthal RS, Albert MA, Buroker AB, Goldberger ZD, Hahn EJ, et al. 2019 ACC/AHA guideline on the primary prevention of cardiovascular disease: a report of the American College of Cardiology/American Heart Association Task Force on Clinical Practice guidelines. Circulation. 2019;140(11):e596–646. 30879355 10.1161/CIR.0000000000000678 PMC7734661

[3] Pirillo A, Casula M, Olmastroni E, Norata GD, Catapano AL. Global epidemiology of dyslipidaemias. Nat Reviews Cardiol. 2021;18(10):689–700. 10.1038/s41569-021-00541-4 33833450

[4] Virani SS, Alonso A, Benjamin EJ, Bittencourt MS, Callaway CW, Carson AP, et al. Heart disease and stroke statistics—2020 update: a report from the American Heart Association. Circulation. 2020;141(9):e139–596. 31992061 10.1161/CIR.0000000000000757

[5] Nakano E, Harada T, Akashi K, Ishizu T, Seo Y, Aonuma K. Impacts of aggressive treatment with statin on cardiovascular events among patients whose LDL-cholesterol concentration within normal range: results from icas registry. Eur Heart J. 2013;34(suppl1):P2275. 10.1093/eurheartj/eht308.P2275

[6] Makover ME, Surma S, Banach M, Toth PP. Eliminating atherosclerotic cardiovascular disease residual risk. Oxford University Press US; 2023. 10.1093/eurheartj/ehad446 37448228

[7] Catapano AL, Barrios V, Cicero AF, Pirro M. Lifestyle interventions and nutraceuticals: Guideline-based approach to cardiovascular disease prevention. Atherosclerosis Supplements. 2019;39:100003. 10.1016/j.athx.2019.100003

[8] Adhyaru BB, Jacobson TA. Safety and efficacy of statin therapy. Nat Reviews Cardiol. 2018;15(12):757–69. 10.1038/s41569-018-0098-5 30375494

[9] Khan SU, Yedlapati SH, Lone AN, Hao Q, Guyatt G, Delvaux N et al. PCSK9 inhibitors and ezetimibe with or without statin therapy for cardiovascular risk reduction: a systematic review and network meta-analysis. BMJ. 2022;377. 10.1136/bmj-2021-069116 35508321

[10] Banach M, Penson PE, Farnier M, Fras Z, Latkovskis G, Laufs U, et al. Bempedoic acid in the management of lipid disorders and cardiovascular risk. 2023 position paper of the international lipid Expert Panel (ILEP). Prog Cardiovasc Dis. 2023;79:2–11. 36889490 10.1016/j.pcad.2023.03.001

[11] Alvarez-Jimenez L, Morales-Palomo F, Moreno-Cabañas A, Ortega JF, Mora-Rodríguez R. Effects of statin therapy on glycemic control and insulin resistance: a systematic review and meta-analysis. Eur J Pharmacol. 2023:175672. 10.1016/j.ejphar.2023.175672 36965747

[12] Banach M, Stulc T, Dent R, Toth PP. Statin non-adherence and residual cardiovascular risk: there is need for substantial improvement. Int J Cardiol. 2016;225:184–96. 27728862 10.1016/j.ijcard.2016.09.075

[13] Bytyçi I, Penson PE, Mikhailidis DP, Wong ND, Hernandez AV, Sahebkar A, et al. Prevalence of statin intolerance: a meta-analysis. Eur Heart J. 2022;43(34):3213–23. 35169843 10.1093/eurheartj/ehac015 PMC9757867

[14] Gallego-Colon E, Daum A, Yosefy C, Statins. PCSK9 inhibitors: a new lipid-lowering therapy. Eur J Pharmacol. 2020;878:173114. 32302598 10.1016/j.ejphar.2020.173114

[15] MacDougall DE, Baum SJ, Ahmed CD, McGowan MP, Wilemon KA. Trends in Patient Access to and Utilization of Prescribed PCSK9 Inhibitors in a Large US Claims Database From 2015 to 2021. Circulation: Cardiovascular Quality and Outcomes. 2024:e009988. 10.1161/CIRCOUTCOMES.123.009988 PMC10880926 38362767

[16] Morton JI, Marquina C, Lloyd M, Watts GF, Zoungas S, Liew D, et al. Lipid-lowering strategies for primary prevention of coronary heart disease in the UK: a cost-effectiveness analysis. PharmacoEconomics. 2024;42(1):91–107. 37606881 10.1007/s40273-023-01306-2 PMC10791963

[17] De Filippo O, D’Ascenzo F, Iannaccone M, Bertaina M, Leone A, Borzillo I, et al. Safety and efficacy of bempedoic acid: a systematic review and meta-analysis of randomised controlled trials. Cardiovasc Diabetol. 2023;22(1):324. 38017541 10.1186/s12933-023-02022-z PMC10685600

[18] Leslie K, McCowan C, Pell J. Adherence to cardiovascular medication: a review of systematic reviews. J Public Health. 2019;41(1):e84–94. 10.1093/pubmed/fdy088 PMC6459362 29850883

[19] Lloyd-Jones DM, Braun LT, Ndumele CE, Smith SC Jr, Sperling LS, Virani SS, et al. Use of risk assessment tools to guide decision-making in the primary prevention of atherosclerotic cardiovascular disease: a special report from the American Heart Association and American College of Cardiology. Circulation. 2019;139(25):e1162–77. 30586766 10.1161/CIR.0000000000000638

